# Relationship Between Clinical Outcomes and Nerve Conduction Studies Before and After Viral Infections in Healthy Individuals: Case Series

**DOI:** 10.7759/cureus.48980

**Published:** 2023-11-17

**Authors:** Sarah H Al-Mazidi, Fawzia ALRouq, Areej S Alsabty, Abdullah Alhajlah, Asma AlYahya, Ahmed Alsabih, Reema Al-taweraqi, Abdullah S Alahmari, Lina Al-Dakhil, Syed Habib

**Affiliations:** 1 Physiology, Imam Mohammad Ibn Saud Islamic University, Riyadh, SAU; 2 Physiology, King Saud University, College of Medicine, Riyadh, SAU; 3 College of Medicine, Imam Mohammad Ibn Saud Islamic University, Riyadh, SAU; 4 Neurology, King Abdulaziz Medical City, Riyadh, SAU; 5 Research, King Saud Medical City, Research Center, Riyadh, SAU; 6 Physiology, King Saud University, Riyadh, SAU

**Keywords:** coronavirus, electrodiagnostic, neurological manifestations, nerve conduction studies, viral infection

## Abstract

Background: The neurological effect of viral respiratory infections has been acknowledged in many studies. However, patients who recovered from this infection show neurological manifestations and are not being routinely transferred for electrodiagnostic evaluation.

Aim: This study aimed to examine the neurological effect of viral respiratory infections on the nerve function using electrophysiology in patients fully recovered from viral respiratory infections.

Methods: To limit bias in the results, the authors decided to choose patients who recovered from one virus in all participants (coronavirus). Medical records were screened for patients who performed nerve conduction studies (NCSs) before the coronavirus pandemic. Thirty patients met our inclusion criteria, and only 10 showed up to perform NCS. Data of the NCS was compared before and after the coronavirus infection for motor and sensory NCS parameters.

Results: An increase in both the median and ulnar sensory nerve latencies and a decrease in the sensory nerve amplitude was observed. Also, there was a decrease in the motor conduction velocity (MCV) of the ulnar nerves and motor amplitude in the median nerve. In the lower limbs, there was a decrease in the sural nerve latency, increased MCV in the tibial nerves, and decreased MCV in the peroneal nerves. The proximal amplitudes of the tibial and peroneal nerves were increased, but the distal amplitude was increased only in the peroneal nerves and decreased in the tibial nerves.

Conclusion: There is a significant impact of viral infections on the peripheral nerves. Large-scale prospective studies are required to investigate the pathogenesis of the neuropathy and myopathy after viral infections.

## Introduction

Central and peripheral neurological manifestations associated with respiratory viral infections are common [1]. The most reported peripheral neurological symptoms in respiratory viral infection patients are myalgia and loss of smell and/or taste [2,3]. Many etiopathogenes of these neurological symptoms were suggested, including neuroinflammation. It has been reported that neuroinflammation caused by the direct effect of the virus leads to nerve injuries and has a central role in these neurological manifestations [4,5]. The severity of the neurological symptoms in viral infections was also linked to the inflammatory profile of patients with neurological symptoms compared to patients without neurological symptoms [6].

There is mounting evidence of the possible neuroinvasive capacity of viral infections, particularly those resulting from coronavirus. Many studies reported abnormalities in nerve conduction studies (NCSs) in patients who recovered from coronavirus infections. Still, these patients showing neurological symptoms were not routinely referred for electrophysiological evaluation. Studies reported that most cases who underwent NCSs presented with myopathy, and a limited number of patients presented with polyneuropathy. As a result, motor NCS and electromyography (EMG) were abnormal in these patients compared to sensory NCS [7]. 

An NCS is a non-invasive electrophysiological diagnostic procedure that evaluates motor and peripheral sensory nerves and allows clinicians to follow the prognosis of the nerve function. It is time efficient and routinely used in the neurophysiology clinic for large peripheral nerves but not small peripheral nerves, which is considered a limitation of the procedure [8].

Results of many case studies and few clinical studies showed inconclusive results regarding the effect of viral infections on the peripheral nerves. These controversial results might be because of the small sample size, medications used during NCSs such as corticosteroids, and confounding factors such as intensive care unit-acquired weakness and pre-existing polyneuropathies. 

To overcome these factors in our study, we investigated the changes in NCSs before and after exposure to a viral infection.

## Materials and methods

This observational study was conducted at the clinical physiology department of King Abdulaziz University Hospital, Riyadh, from March 2022 to November 2022.

Procedure

Medical records of patients who underwent NCSs before January 2020 were reviewed. To limit bias, we chose one particular respiratory viral infection, i.e., coronavirus. The medical records of 50 patients were initially included according to our selection criteria. Of these patients, 30 were found to be infected with coronavirus within the past 12 months, and only 10 have accepted to participate and came for NCS re-evaluation. The participants' demographics included age, sex, occupation, medical history, infection history, and symptoms (past and current) associated with the viral infection. 

Study participants

The participants included adults (≥18 of age), both males and females (six and four, respectively) who underwent NCSs before the coronavirus pandemic. All patients had a confirmed infection by real-time polymerase chain reaction (PCR) of nasopharyngeal swabs and were not hospitalized during their illness. 

Patients with known neuropathy, myopathy, neurological disorders, such as stroke and multiple sclerosis, and active infections in the past three months were excluded from the study.

Ethical considerations

All participants provided written informed consent according to the Declaration of Helsinki. The Institutional Review Board of the College of Medicine at King Saud University approved the study protocol for human research (IRB approval no. E-22-6646, dated February 8, 2022).

NCSs

NCSs include sensory and motor nerve conduction studies and F-wave responses. We followed the American Society of Electromyography, American Academy of Neurology, and American Academy of Physical Medicine and Rehabilitation as guidelines for the NCSs. The NCSs were performed for the median and ulnar nerves in the upper limbs and the lower limbs' sural, tibial, and peroneal nerves using the Nicolet machine (Nicolet EDX with Natus Elite Software, Orlando, Florida, USA). Parameters recorded were motor nerve study (MNS) parameters, including motor nerve conduction velocity (MNCV), compound muscle action potential amplitude (CMAP) distal and proximal latency, and amplitude. Sensory nerve studies (SNSs) were conducted by an antidromic stimulation. Measurements were made of orthodromic sensory nerve conduction from the index, middle, and little fingers to the wrist, with surface recordings made over the median proximal to the distal wrist crease. The SNS parameters were sensory nerve action potential (SNAP) latency, nerve conduction velocity (SNVC), and amplitude. Values of NCSs were recorded before and after the viral infection.

The type of tested nerves in our study depends on the tested nerves prior to the viral infection nerve evaluation for each participant.

Statistical analysis

The Stata Statistical Software (release 13, StataCorp., 2023, College Station, TX: StataCorp LLC) was used for the data analysis. Categorical variables were presented as frequency, while continuous variables were presented as median and interquartile.

## Results

Demographics

A total of 10 cases underwent detailed NCSs. Our sample included six male and four female participants (Table 1). Seven patients underwent upper limb NCS, and three underwent lower limb NCS. A total of 14 median and ulnar nerves and six sural, tibial, and peroneal nerves were assessed. The mean age of the participants was 50.6 (range 30-68). The mean duration from diagnosis to electrophysiological assessment was 9.3 ± 3.7 months. 

**Table 1:** Demographics of the participants Values are expressed as median and interquartile range (IQR). BMI = body mass index

Variable	Median (IQR)
Gender (M\F)	Female (4) Male (6)
Age (years)	54 (16)
Height (cm)	168 (23)
Weight (Kg)	75 (35)
BMI	29 (20)

**Figure 1:**
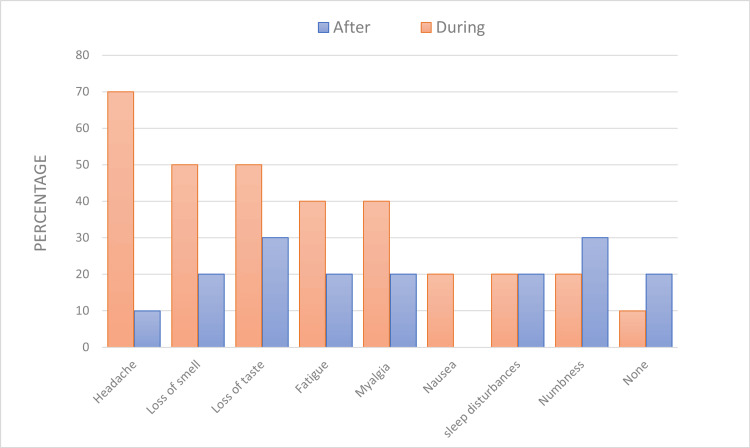
Proportion of the clinical features of the participants during and after the viral infection Data are presented as percentage (%).

Figure 1 shows the clinical features of the participants during and after the viral infection. The frequently reported symptoms were headache (70%, N = 7), loss of taste and smell (50%, N = 5), and myalgia (40%, N = 4). These symptoms persisted in 60% (N = 6) of the participants. 

NCSs

Table 2 and Table 3 demonstrate NCSs, including both sensory and motor nerve conduction parameters in the upper limbs for each case. We observed an increase in the SNAP latency in the median and ulnar nerves. The SNAP amplitude was decreased in both the ulnar and median nerves (more decreased for the median nerve). MCV was decreased in both the median and ulnar nerves. Moreover, a reduced CMAP amplitude was observed in the median nerves.

**Table 2:** Nerve conduction studies of the upper limbs before and after the viral infection SNAP = sensory nerve action potential, SNVC = sensory nerve conduction velocity, MNS = motor nerve studies, CV = conduction velocity, PP = peak to peak, Amp = amplitude, B = before, A = after Data are presented as numbers.

Site	Upper limbs											
Case	Case 1				Case 2				Case 3			
Side	Right		Left		Right		Left		Right		Left	
Gender	Male				Male				Male			
Age	50				58				58			
Time	Before	After	Before	After	Before	After	Before	After	Before	After	Before	After
Median												
SNAP (µvol)	2.76	3.23	2.86	3.23	3.7	4.43	3.91	5.85	2.76	3.28	3.94	3.33
SNCV (m/sec)	51	43	40	43	38	32	36	36	51	43	30	42
MNS												
Distal amplitude (mvol)	5.8	5.3	3.23	3.02	11.8	8.1	3.18	3.65	5.11	6.3	2.71	2.92
Proximal amplitude (mvol)	4.3	1.4	7.71	7.29	11.4	12.3	7.5	7.97	6.7	6.6	8.49	7.71
CV m/sec	52	53	58	56	58	54	58	51	53	54	50	52
F-wave (ms)	30.4	27.1	28.2	26.7	28.6	31.5	28.9	29.2	31.5	29.2	30.4	28.8
Amplitude												
Sensory P.P. (µvol)	102.8	45.1	102.8	93.5	54.7	31.8	54.8	49.7	107	66.4	110.6	73.8
Motor distal (mvol)	2.81	3.33	9.6	8.2	3.94	4.84	9	14.5	2.76	3.02	10.8	13.2
Motor proximal (mvol)	7.97	7.45	4.7	1.1	7.81	8.33	8.6	1.7	8.39	7.66	11.2	5
Ulnar												
SNAP (µvol)	2.55	3.07	2.76	3.13	2.76	5.99	3.07	6.93	3.02	3.39	2.81	3.28
SNCV (m/sec)	55	46	51	45	51	23	46	20	46	41	50	43
MNS												
Distal amplitude (mvol)	2.71	2.6	2.19	2.86	2.81	3.91	2.81	3.33	2.45	2.71	2.5	2.81
Proximal amplitude (mvol)	6.41	6.35	6.93	6.46	7.29	8.49	8.54	8.39	6.72	7.24	7.08	7.34
CV m/sec	68	59	55	58	56	41	50	44	66	60	61	53
F-wave (ms)	29	28.4	29.9	26.1	30.8	631.7	32	32.1	30.6	28.2	31.1	29.8
Amplitude												
Sensory P.P. (µvol)	62.6	68.2	36.4	18.6	114.6	38.7	4.4	28.5	58.5	33.7	91.8	70.4
Motor distal (mvol)	10.6	10.1	13.5	12.6	5.8	8.4	8.8	4.8	10.2	7.9	7.9	8.7
Motor proximal (mvol)	11.3	10.8	13.2	13.3	3	5.2	5.7	0.9	10.5	7.5	8.6	7.8

**Table 3:** Nerve conduction studies of the upper limbs before and after the viral infection SNAP = sensory nerve action potential, SNVC = sensory nerve conduction velocity, MNS = motor nerve studies, CV = conduction velocity, PP = peak to peak, Amp = amplitude, B = Before, A = After Data are presented as numbers.

Site	Upper limbs															
Case	Case 4				Case 5				Case 6				Case 7			
Side	Right		Left		Right		Left		Right		Left		Right		Left	
Gender	Female				Female				Female				Female			
Age	58				50				58				58			
Time	Before	After	Before	After	Before	After	Before	After	Before	After	Before	After	Before	After	Before	After
Median																
SNAP (µvol)	2.6	3.39	2.45	3.33	3.44	3.28	3.13	3.18	3.13	3.54	3.18	3.44	2.66	0	2.55	3.18
SNCV (m/sec)	54	41	57	42	41	43	45	44	45	40	44	41	53	0	55	44
MNS																
Distal Amp (mvol)	2.45	2.81	2.5	2.81	3.28	3.13	3.44	2.71	2.92	3.07	2.86	2.97	2.76	3.44	2.92	2.92
Proximal Amp (mvol)	7.03	6.88	5.73	6.88	6.98	6.2	5.73	6.41	2.34	6.98	6.41	6.46	6.25	15.5	7.08	7.08
CV m/sec	55	52	67	52	59	63	58	57	55	50	73	55	63	15	50	50
F-wave (ms)	25.9	27.6	27.1	24.2	25.5	24.8	25	25.8	25.5	26.5	24.3	24.1	26.1	29.8	26.8	26.8
Ulnar																
SNAP (µvol)	2.55	3.18	2.81	4.01	2.4	2.92	2.76	2.86	2.34	2.92	2.55	3.07	2.71	3.49	3.23	3.33
SNCV (m/sec)	55	44	50	35	58	48	51	49	60	48	55	46	52	40	42	42
MNS																
Distal Amp (mvol)	2.14	2.66	2.14	2.4	2,34	2.34	2.08	2.19	2.03	3.07	2.4	3.13	2.4	2.66	2.08	2.71
Proximal Amp (mvol)	5.94	5.47	5.42	5.73	6.09	5.63	5.73	5.47	5.1	10.5	5.83	6.25	5.83	6.35	6.25	6.41
CV m/sec	74	68	76	69	59	58	58	55	72	21	70	69	70	50	67	51
F-wave (ms)	25.3	23.2	24.1	24	25.4	24.2	25	26.7	25.2	25.2	24.8	24	27.7	28	28.2	25.8

**Figure 2:**
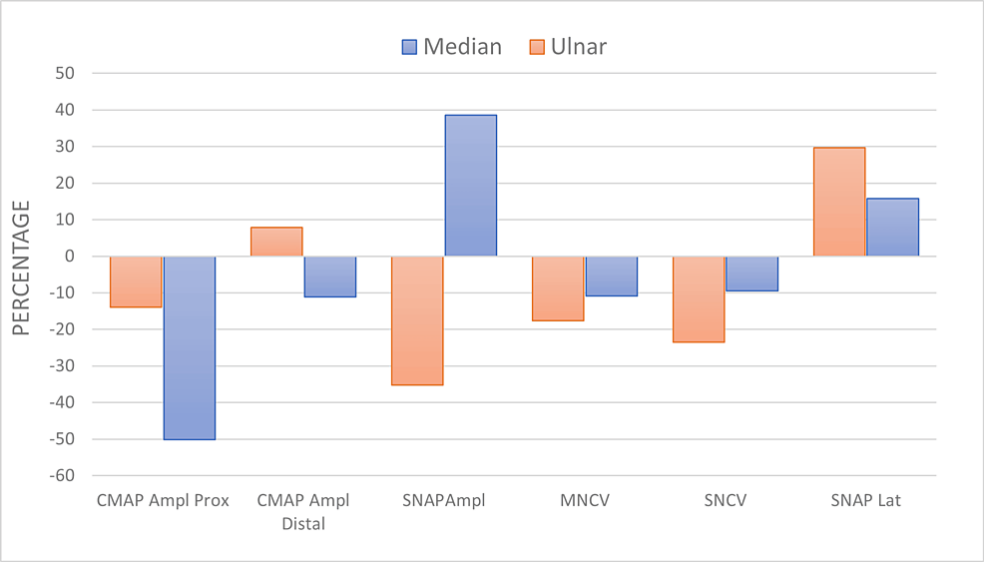
Percentage changes in the NCS parameters in the median and ulnar nerves. SNAP = sensory nerve action potential, SNVC = sensory nerve conduction velocity, MNCV = motor nerve conduction velocity, CMAP Ampl Distal = compound muscle action potential amplitude (distal), CMAP Ampl Prox = compound muscle action potential amplitude (Proximal), NCS = nerve conduction study Data are presented as percentages.  An increase in values after the viral infection are presented as a positive percentage, and a decrease in values after the viral infection are presented as a negative percentage.

The mean percent changes in our participants for the SCV, MCV, SNAP, and CMAP in the upper limbs are presented in Figure 2. The increase in latency (SNAP) in the median nerve was 15.8% (0.53) and in the ulnar nerve was 29.6% (0.94). The decrease in velocity in the median nerve was 9.3% (-4.07) and in the ulnar nerve was 23.5%. The decrease in amplitude in the median nerve was 38.6% (-28.73) and in the ulnar nerve was 35.2% (-18.36).

As for the MNS, the percent increase in amplitude was 6.5% (0.20) for the median nerve and 17.4% (0.49) for the ulnar nerve. The percent decrease in the CV of the median nerve was 10.9% (-5.92) and in the ulnar nerve was 17.6% (-10.42). The percent decrease in motor amplitude for the ulnar nerve was 13.9% (-1.13), and the median was 50.1% (-3.13).

In the lower limbs, detailed NCS parameters are presented in Table 4. When comparing the mean of the results of the NCSs of the lower limbs in our participants, we observed an 18% decrease in the SNAP latency of the sural nerves. 

**Table 4:** Nerve conduction studies of the lower limbs before and after the viral infection CV = conduction velocity Data are presented as numbers.

Site	Upper limbs											
Case	Case 1				Case 2				Case 3			
Side	Right		Left		Right		Left		Right		Left	
Gender	Male				Male				Male			
Age	34				63				42			
Time	Before	After	Before	After	Before	After	Before	After	Before	After	Before	After
Peroneal												
Distal (mvol)	4.84	3.33	4.38	4.64	2.97	3.54	5	5.99	6.93	6.82	5.42	4.27
Proximal (mvol)	11.61	10.78	12.5	12.08	10.1	9.84	11.25	12.34	15.52	14.17	12.86	12.71
CV (m/sec)	53	48	49	48	49	52	56	52	44	43	52	40
F-wave (ms)	45.6	50.5	54.2	53.9	46.5	47	48.3	51.9	52.4	60.7	54.6	55
Tibial												
Distal (mvol)	3.96	3.65	4.38	4.01	5	5.57	5.94	5.36	3.33	3.59	4.64	3.65
Proximal (mvol)	13.28	12.5	14.06	12.66	14.11	10.94	15.1	12.76	13.44	13.75	14.15	12.55
CV (m/sec)	45	47	44	51	50	62	44	43	41	36	41	44
F-wave (ms)	51.9	50.3	55.9	54.3	51.7	46.8	50.1	43	52.5	52	53.9	51.6
Sural												
Latency (ms)	2.71	2.45	3.83	1.98	3.28	2.81	3.49	3.82	3.59	Absent	Absent	Absent
CV (m/sec)	52	53	43	63	49	43	52	37	39	Absent	39.8	Absent

**Figure 3:**
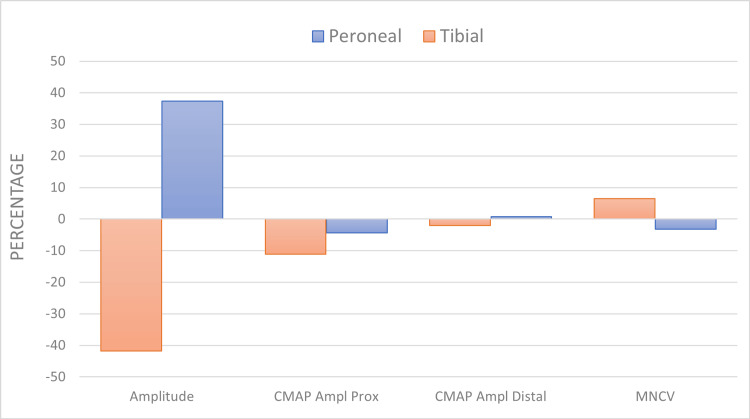
Percentage change in the NCS parameters in the peroneal and tibial nerves. MNCV = motor nerve conduction velocity, CMAP Ampl Distal = compound muscle action potential amplitude (distal), CMAP Ampl Prox = compound muscle action potential amplitude (Proximal) Data are presented as percentages. An increase in values after the viral infection is presented as a positive percentage, and a decrease in values after the viral infection is presented as a negative percentage.

Moreover, an increase in MCV in the tibial nerve and a decrease in the peroneal MCV was observed (Figure 3). The proximal amplitudes of the tibial and peroneal nerves were increased, but the distal amplitude was increased only in the peroneal nerves and decreased in the tibial nerves. 

## Discussion

This study assessed the pre- and post-exposure of a viral infection in normal individuals using NCSs. We found that there are changes in NCS parameters in individuals who recovered from the viral infection. 

The most common clinical neurological symptoms in our participants were fatigue and myalgia, which were reported by 40% [9]. One of the most prevalent clinical symptoms in our study was headache. Headache affects half of coronavirus patients and is more frequent in outpatients than hospitalized patients [10]. Moreover, a study reported that headaches persisted for up to nine months after recovering from a coronavirus infection [11]. These reports support our results since all our patients were outpatients, and some complained of headaches after nine months of recovery. Many pathogeneses were suggested for headaches in coronavirus patients, such as direct viral effects and inflammation, but these outcomes were inconclusive [10].

The neurological effect of viral infections has been reported resulting in myopathy and central and peripheral neuropathies. Studies on the peripheral impact of coronavirus infections mainly reported neuropathy of muscular origin, but others reported that it also affected the peripheral nerves resulting in peripheral neuropathy [12]. Our results showed that both sensory and motor parameters were affected. The effect of the viral infection on our participants was scattered and not uniform, which agrees with a previous report [13]. The reason for this ununiform effect of the virus in the NCS results has been explained in many studies as theories with no definite conclusion specifically for the coronavirus infection. The most relevant etiology is the cytokine storm associated with coronavirus, which might affect individuals differently. Moreover, myalgia and sleep disturbances were persistent in coronavirus patients for up to six months post-infection, supporting our results [14]. 

We observed that the impact of the viral infection on the amplitude was higher than the CV, which implies that the myeline sheet and axons may be affected by the virus. Previous reports also support our findings [13]. Central and peripheral demyelinations are well known in para- and post-viral infection [15]. Studies reported that coronavirus has an acute and delayed post-infection effect on nerve demyelination in the central nervous system. Reports showed that demyelination could be caused by a direct viral invasion or immune response demyelination [16]. 

Although the values of NCSs in our study fall within the normal ranges, the changes observed from the baseline were significant. The presentation of NCS parameters was patchy; for example, percent change was significantly affected in the SNAP amplitude of the upper limb, but the CV was only considerably changed in the ulnar nerve. Moreover, the proximal amplitude was affected in the median and tibial nerves but distal in the ulnar nerve. Previous studies also showed normal values of sensory NCS with minor changes observed in the motor NCS in coronavirus patients suggesting a myopathy rather than sensory neuropathy [17,18]. These findings were also affected by hospitalization and the severity of the disease, which might develop later after recovery [12,19].

Electrodiagnostic studies are not routinely performed in coronavirus patients with neurological symptoms [12]. Previous studies mainly reported a normal NCS in coronavirus patients, which is in agreement with our results [7]. However, these reports were based on case studies and series with major variations in patient selection criteria. It was difficult in these studies to rule out the confounding factors that might affect NCS results, such as medications, pre-existing neurological diseases, and the effect of intensive care-acquired weakness. To overcome these factors, we excluded cases with pre-existing neurological disorders and hospitalized patients.

The NCS is a valid electrodiagnostic tool routinely used in neurophysiology clinics. It objectively determines the functions of the peripheral nerves. In neuromuscular disorders, the long and peripheral nerves are the most affected [20]. The NCS can be used as a diagnostic tool to determine patients' prognoses [21]. It also can predict recovery outcomes in neurological conditions [22]. One of the disadvantages of routine NCS is that it cannot test for small nerve fiber neuropathy [23]. Many factors have a role in determining the average reference values of NCSs. Demographics, including age, gender, BMI, and height, are the most common factors that alter NCS values [24]. Ethnicity is another factor identified, which could affect NCS values [25,26]. The frequently used reference values for NCSs were established by the American Association of Neuromuscular and Electrodiagnostic Medicine, which is not universally accepted in all ethnic groups [27]. The NCS normal values in different countries were examined, showing diversity in NCS normal ranges, which suggests a redefinition of these normative references in consideration of all the factors mentioned above [28]. Moreover, the side-to-side difference in NCS values was determined [26]. Our study compared the NCS values in the same patient with a maximum of two years' differences between pre- and post-viral infection. This eliminates all factors that might affect the results of NCSs in our tested samples. 

In the present study, the comparative values of the pre- and post-infections were different in the median and ulnar nerves. This difference might indicate the neuropathic effects of the virus on the upper limb nerves regardless of normal values obtained from our participants. A standardized electrodiagnostic approach for patients with a respiratory viral infection and showing neurological manifestation is crucial in determining the neuromuscular outcomes in these patients. This recommendation is supported by a study stating that a NCS was a significant determinant of neuromuscular rehabilitation outcomes in coronavirus patients [19].

In-depth studies are needed on the neurotoxic effect of viral infections, and experimental studies on animal models are necessary to explore the pathogenic mechanism underlying these observations. Also, patients at any severity level who show neurological clinical features need a detailed neurological examination supported by both nerve conduction and needle electromyographic studies. Moreover, we need studies on the impact of respiratory viral infections on central neuropathy.

Limitations

 Our study's limitations include the small sample size. Because of the limited clinical studies on the effect of coronavirus disease on the peripheral nerves, the comparison of our results to the available literature was based on case studies and case series, which might present heterogeneity in data collection.

## Conclusions

Our study concludes that viral infections have a significant effect on the peripheral nerves and may cause slowly progressing neuropathy in such cases. Therefore, close follow-up, management strategies, and standardized electrodiagnostic guidelines are recommended to be established for the early diagnosis of neural functions to ensure optimal recovery. Large-scale prospective studies are required to explore the pathogenic mechanisms that lead to neuropathic and myopathic involvements after viral infections.
